# Analysis of factors associated with depressive symptoms in stroke patients based on a national cross-sectional study

**DOI:** 10.1038/s41598-024-59837-3

**Published:** 2024-04-23

**Authors:** Wenhui Xiao, Ying Liu, Jinglin Huang, Li-an Huang, Ying Bian, Guanyang Zou

**Affiliations:** 1 State Key Laboratory of Quality Research in Chinese Medicine, Institute of Chinese Medical Sciences, University of Macau, Taipa, Macau SAR China; 2 Department of Public Health and Medicinal Administration, Faculty of Health Sciences, University of Macau, Taipa, Macau SAR China; 3 School of Public Health and Management, Guangzhou University of Chinese Medicine, Guangzhou, China; 4 School of Public Health, Zhejiang University, Hangzhou, China; 5 The First Affiliated Hospital, Jinan University, Guangzhou, China

**Keywords:** Comorbidities, Health policy, Public health, Stroke

## Abstract

Post-stroke depression is commonly experienced by stroke survivors and has a significant negative impact on the physical, cognitive, and social functioning of those affected. This study aims to investigate the prevalence of depressive symptoms and their associated factors in Chinese stroke patients. Research samples were selected from the China Health and Retirement Longitudinal Study 2018 survey. Depression was evaluated using the 10-item Center for Epidemiological Studies Depression Scale, with a score ≥ 10 defined as depression. Univariate and multivariable analyses were performed to examine the associations of depressive symptoms with demographics, family relationships, health status, and lifestyle. A total of 963 stroke patients were included and 57.8% of them had depressive symptoms. Depressive symptoms were significantly associated with female sex (OR 1.762, 95% CI 1.235–2.514), lower education level (non-formal education: OR 2.148, 95% CI 1.235–3.737, primary to secondary school education: OR 1.964, 95% CI 1.272–3.033), dissatisfaction with spouse (OR 1.912, 95% CI 1.075–3.401), dissatisfaction with life (OR 1.779, 95% CI 1.080–2.931), dissatisfaction with health (OR 1.592, 95% CI 1.138–2.226), pain (OR 1.392, 95% CI 1.005–1.928) and abnormal sleep (OR 1.557, 95% CI 1.126–2.152). The findings suggest the need for regular depression screening and evaluation after a stroke, and that a well-functioning support system, effective health management, and lifestyle modifications could potentially improve the mental state of stroke patients.

## Introduction

A stroke is a medical emergency that occurs when the blood supply to the brain is blocked by a clot or when a blood vessel in the brain ruptures. This can result in lasting brain damage, long-term disability, and even death for the individual. Globally, it was estimated that 143 million years of healthy life were lost due to stroke-related death and disability in 2019, accounting for 5.7% of the Disability-Adjusted Life Years (DALYs)^1^. Stroke was the leading cause of death in China^2^. There were 3.94 million new stroke cases and 2.19 million deaths attributed to stroke in 2019^3^. Moreover, the burden of stroke in China has been steadily increasing over the past few years. According to a large-scale, population-based study covering 4,229, 616 Chinese adults aged 40 years and above, the prevalence of stroke increased by 13.2% from 2013 to 2019^4^.

Post-stroke depression is a common neuropsychiatric complication experienced by stroke survivors^5^. It is characterized by persistent feelings of sadness, hopelessness, and loss of interest in activities that were once enjoyed^6^. Studies have suggested that about one-third of stroke survivors develop depression after a stroke onset^5,7,8^. Compared with post-stroke patients without depression, individuals with post-stroke depression have more pronounced cognitive impairment, lower quality of life, worse rehabilitation outcomes, and a higher risk of mortality^7,9–11^. Post-stroke depression has significant negative impacts on the physical, cognitive, and social functioning of stroke survivors, not only affecting the individual recovery and well-being but also adding a great burden on their families and society^12,13^. Early recognition and effective management of depression in stroke patients are crucial to prevent adverse outcomes and improve rehabilitation potential.

Post-stroke depression has multiple biological, psychological, and social risk factors. Age and sex were the most studied risk factors for post-stroke depression, however, conclusions about the impact of these factors on post-stroke depression remained controversial^5,14^. Several studies identified stroke-related characteristics such as stroke type, stage, severity, and lesion location as important predictors of post-stroke depression^15–17^. Other factors associated with post-stroke depression included impaired cognition, functional dependence, physical pain, and inability to work^18–20^. While previous research has primarily focused on the general and stroke-related factors as determinants of post-stroke depression, understanding patients' modifiable lifestyle and behaviors is equally important to reduce the risk of post-stroke depression. According to Vermeer et al., smoking and receiving stroke treatment were significantly associated with depressive symptoms in stroke survivors^21^. Ayerbe et al. reported an association between low activity levels and post-stroke depression^20^. Social support is another critical factor in combating post-stroke depression^5^, and family relationships are an integral part of social support, especially for Chinese elderly^22^. A longitudinal study by Wang et al. verified that good family functioning was an important protective factor against post-stroke depression at different phases of stroke^23^. However, it is important to note that evidence regarding the association between personal lifestyle, family relationships, and post-stroke depression remains limited, particularly in China. Therefore, the objective of this study is to determine the prevalence of depressive symptoms and their associations with demographics, family relationships, health status, and lifestyle in Chinese stroke patients.

## Results

### Demographic characteristics and depressive symptoms of stroke patients

Among the 963 stroke patients, the mean CESD-10 score was 11.3 points, with 557 individuals (57.8%) exhibiting depressive symptoms. Most patients were aged 60 years or older (74.2%), had primary to secondary education (65.9%), were married (77.5%), lived in rural areas (67.7%), and about half were male (54.5%).

The univariate analysis showed that sex and education level were significantly associated with depressive symptoms in stroke patients. Female participants were more likely to report depressive symptoms than male participants (68.5% vs. 49.0%, *P* < 0.05). Patients with non-formal education, and with primary to secondary school education had a higher proportion of depressive symptoms than those with high school and above education (69.4% vs. 58.6% vs. 37.1%, *P* < 0.05) (Table 1).

**Table 1:** Demographic characteristics and depressive symptoms of stroke patients in China.

Variable	Total	Depressive	Symptoms	*t or χ*^*2*^	*P*-value
		Yes	No		
Total	963	557 (57.8)	406 (42.2)		
Age (mean, range)	65.0 (46–89)	64.6 (46–89)	65.7 (47–86)	− 1.883	0.060
Age (n, %)				1.631	0.202
45~	248 (25.8)	152 (61.3)	96 (38.7)		
60~	715 (74.2)	405 (56.6)	310 (43.4)		
Sex (n, %)				36.594	< 0.001
Male	525 (54.5)	257 (49.0)	268 (51.0)		
Female	438 (45.5)	300 (68.5)	138 (31.5)		
Education level (n, %)				34.099	< 0.001*
High school and above	132 (13.7)	49 (37.1)	83 (62.9)		
Primary to secondary school	635 (65.9)	372 (58.6)	263 (41.4)		
No formal education	196 (20.4)	136 (69.4)	60 (30.6)		
Marital status (n, %)				2.196	0.160
Married	746 (77.5)	422 (56.6)	324 (43.4)		
Single/divorced/widowed	217 (22.5)	135 (62.2)	82 (37.8)		
Place of residence (n, %)				4.783	0.091
Urban	223 (23.2)	121 (54.3)	102 (45.7)		
Mixed zone	88 (9.1)	44 (50.0)	44 (50.0)		
Rural	652 (67.7)	392 (60.1)	260 (39.9)		

*There is statistical significance between ‘high school and above’ and ‘primary to secondary school’, between ‘high school and above’ and ‘no formal education’, and between ‘primary to secondary school’ and ‘no formal education’.

### Family relationships and depressive symptoms of stroke patients

Most of the patients had 1–2 children (67.4%) and were cared for by family members (69.9%). The vast majority of patients were satisfied with their children (93.6%), 76.7% were satisfied with their spouse, and 84.3% were satisfied with their life.

The univariate analysis showed that family care, relationships with children and spouses, and life satisfaction were significantly associated with depressive symptoms in stroke patients. Patients without family caregivers had a higher proportion of depressive symptoms than those with (63.4% vs. 55.4%, *P* < 0.05). Patients who were dissatisfied with their children were more likely to have depressive symptoms than those who were satisfied (81.5% vs. 56.2%, *P* < 0.05). The proportion of depressive symptoms was higher in patients who were dissatisfied with their spouse than that of those who were satisfied with their spouse (80.8% vs. 53.7%, *P* < 0.05), and who did not have a spouse (80.8% vs. 64.0%, *P* < 0.05). Compared with patients who were satisfied with their life, patients who were dissatisfied were more likely to have depressive symptoms (80.8% vs. 53.6%, P < 0.05) (Table 2).

**Table 2:** Family relationships and depressive symptoms of stroke patients in China.

Variable	Total	Depressive	Symptoms	*χ*^*2*^	*P*-value
		Yes	No		
Total	963	557 (57.8)	406 (42.2)		
Number of children (n, %)				0.655	0.418*
3~	306 (31.8)	182 (59.5)	124 (40.5)		
1–2	649 (67.4)	368 (56.7)	281 (43.3)		
0	8 (0.8)	7 (87.5)	1 (12.5)		
Family care (n, %)				5.352	0.021
Yes	673 (69.9)	373 (55.4)	300 (44.6)		
No	290 (30.1)	184 (63.4)	106 (36.6)		
Relationships with children (n, %)				13.375	< 0.001^**†**^
Satisfied	901 (93.6)	506 (56.2)	395 (43.8)		
Dissatisfied	54 (5.6)	44 (81.5)	10 (18.5)		
No child	8 (0.8)	7 (87.5)	1 (12.5)		
Relationships with spouse (n, %)				28.503	< 0.001^**‡**^
Satisfied	739 (76.7)	397 (53.7)	342 (46.3)		
Dissatisfied	99 (10.3)	80 (80.8)	19 (19.2)		
No spouses	125 (13.0)	80 (64.0)	45 (36.0)		
Life satisfaction (n, %)				38.695	< 0.001
Satisfied	812 (84.3)	435 (53.6)	377 (46.4)		
Dissatisfied	151 (15.7)	122 (80.8)	29 (19.2)		

*The group ‘0’ were not included in the chi-square test.

^**†**^The group “no child” were not included in the chi-square test.

^**‡**^There is statistical significance between ‘satisfied’ and ‘dissatisfied’, between ‘dissatisfied’ and ‘no spouses’.

### Health status and depressive symptoms of stroke patients

Regarding health status, half of patients (52.9%) rated their health as being poor while only 8.6% rated their health as being good. Half of patients (50.5%) were satisfied with their health and half (49.5%) were not. The majority of patients (79.1%) had two or more co-morbid conditions. A total of 68.3%, 26.3%, 38.9% and 17.2% had hypertension, diabetes mellitus, heart disease, and lung disease, respectively. Most of the patients (69.8%) suffered from physical pain, and 39.1% were able to work normally.

The univariate analysis showed that self-rated health, health satisfaction, co-morbid conditions, heart disease, lung disease, physical pain, and work ability were significantly associated with depressive symptoms in stroke patients. Specifically, the proportion of depressive symptoms was higher in patients with poor self-rated health than that of those with fair (66.2% vs. 50.9%, P < 0.05) and good self-rated health (66.2% vs. 37.3%, P < 0.05). Patients who were dissatisfied with their health were more likely to have depressive symptoms than patients who were satisfied (69.6% vs. 46.3%, P < 0.05). Patients with two or more co-morbid conditions had a higher proportion of depressive symptoms than those with only one (61.4% vs. 44.4%, P < 0.05) and without (61.4% vs. 44.0%, P < 0.05). Patients who had heart disease (64.8% vs. 53.4%, P < 0.05), lung disease (66.9% vs. 56.0%, *P* < 0.05), and physical pain (64.3% vs. 43.0%, P < 0.05) were more likely to experience depressive symptoms. Patients who were unable to work long hours (65.8% vs. 47.7%, P < 0.05) and work at all (62.6% vs. 47.7%, P < 0.05) had a higher proportion of depressive symptoms compared to those who were able to work normally (Table 3).

**Table 3:** Health status and depressive symptoms of stroke patients in China.

Variable	Total	Depressive	Symptoms	*Χ*^*2*^	*P*-value
		Yes	No		
Total	963	557 (57.8)	406 (42.2)		
Self-rated health (n, %)				36.144	< 0.001*
Good	83 (8.6)	31 (37.3)	52 (62.7)		
Fair	371 (38.5)	189 (50.9)	182 (49.1)		
Poor	509 (52.9)	337 (66.2)	172 (33.8)		
Health satisfaction (n, %)				53.618	< 0.001
Satisfied	486 (50.5)	225 (46.3)	261 (53.7)		
Dissatisfied	477 (49.5)	332 (69.6)	145 (30.4)		
Co-morbid conditions (n, %)				19.160	< 0.001^**†**^
0	50 (5.2)	22 (44.0)	28 (56.0)		
1	151 (15.7)	67 (44.4)	84 (55.6)		
2~	762 (79.1)	468 (61.4)	294 (38.6)		
Hypertension (n, %)				0.132	0.717
No	305 (31.7)	179 (58.7)	126 (41.3)		
Yes	658 (68.3)	378 (57.4)	280 (42.6)		
Diabetes mellitus (n, %)				0.976	0.323
No	710 (73.7)	404 (56.9)	306 (43.1)		
Yes	253 (26.3)	153 (60.5)	100 (39.5)		
Heart disease (n, %)				12.200	< 0.001
No	588 (61.1)	314 (53.4)	274 (46.6)		
Yes	375 (38.9)	243 (64.8)	132 (35.2)		
Lung disease (n, %)				6.703	0.010
No	797 (82.8)	446 (56.0)	351 (44.0)		
Yes	166 (17.2)	111 (66.9)	55 (33.1)		
Physical pain (n, %)				37.888	< 0.001
No	291 (30.2)	125 (43.0)	166 (57.0)		
Yes	672 (69.8)	432 (64.3)	240 (35.7)		
Work ability (n, %)				26.494	< 0.001^**‡**^
Work normally	377 (39.1)	180 (47.7)	197 (52.3)		
Unable to work long hours	313 (32.5)	206 (65.8)	107 (34.2)		
Unable to work at all	273 (28.3)	171 (62.6)	102 (37.4)		

*There is statistical significance between ‘good’ and ‘poor’, between ‘fair’ and ‘poor’; ^**†**^ There is statistical significance between ‘0’ and ‘2 ~ ’, between ‘1’ and ‘2 ~ ’; ^**‡**^ There is statistical significance between ‘work normally’ and ‘unable to work long hours’, between ‘work normally’ and ‘unable to work at all ’.

### Lifestyle and depressive symptoms of stroke patients

Most stroke patients did not drink alcohol (70.8%), did not smoke (74.7%), and had physical activities (86.9%), while only half (54.2%) participated in social activities. Most of the patients (70.2%) had normal sleep, and 69.3% were receiving stroke treatment.

The univariate analysis showed that patients with abnormal sleep had a higher proportion of depressive symptoms than those with normal sleep (69.3% vs. 53.0%, *P* < 0.05). Patients who were under stroke treatment were more likely to have depressive symptoms than those who were not (60.3% vs. 52.4%, *P* < 0.05) (Table 4).

**Table 4:** Lifestyle and depressive symptoms of stroke patients in China.

Variable	Total	Depressive	Symptoms	*χ*^*2*^	*P*-value
		Yes	No		
Total	963	557 (57.8)	406 (42.2)		
Drinking (n, %)				3.773	0.052
No	682 (70.8)	408 (59.8)	274 (40.2)		
Yes	281 (29.2)	149 (53.0)	132 (47.0)		
Smoking (n, %)				3.312	0.069
No	719 (74.7)	428 (59.5)	291 (40.5)		
Yes	244 (25.3)	129 (52.9)	115 (47.1)		
Physical activity (n, %)				0.636	0.425
Yes	837 (86.9)	480 (57.3)	357 (42.7)		
No	126 (13.1)	77 (61.1)	49 (38.9)		
Social activity (n, %)				0.147	0.702
Yes	522 (54.2)	299 (57.3)	223 (42.7)		
No	441 (45.8)	258 (58.5)	183 (41.5)		
Sleep duration (n, %)				22.165	< 0.001
Normal	676 (70.2)	358 (53.0)	318 (47.0)		
Abnormal	287 (29.8)	199 (69.3)	88 (30.7)		
Stroke treatment (n, %)				5.254	0.022
Yes	667 (69.3)	402 (60.3)	265 (39.7)		
No	296 (30.7)	155 (52.4)	141 (47.6)		

### Multi-variable analysis of depressive symptoms of stroke patients

The regression analysis further confirmed the significant associations between sex, educational level, relationships with spouse, life satisfaction, health satisfaction, physical pain, sleep duration, and depressive symptoms. Females were more likely to have depressive symptoms than males (OR 1.762, 95% CI 1.235–2.514). The probability of having depressive symptoms in patients with non-formal education and primary school to secondary school education was found to be 2.148 times (95% CI 1.235–3.737) and 1.964 times (95% CI 1.272–3.033) higher than that of patients with high school education and above, respectively. Patients who were not satisfied with their spouse (OR 1.912, 95% CI 1.075–3.401) and life (OR 1.779, 95% CI 1.080–2.931) had a higher likelihood of experiencing depressive symptoms than those who were. Health dissatisfaction (OR 1.592, 95% CI 1.138–2.226) and physical pain (OR 1.392, 95% CI 1.005–1.928) were also significantly associated with depressive symptoms. Patients with abnormal sleep tended to have depressive symptoms compared with those with normal sleep (OR 1.557, 95% CI 1.126–2.152) (Table 5).

**Table 5:** Multivariable analysis of depressive symptoms of stroke patients in China.

Variable	β	SE	Wald	*P-*value	OR	95% CI
Demographics						
Sex: female	0.566	0.181	9.762	0.002	1.762	1.235–2.514
Education: primary to middle school	0.675	0.222	9.276	0.002	1.964	1.272–3.033
Education: no formal education	0.765	0.282	7.336	0.007	2.148	1.235–3.737
Marital status: single/divorced/widowed	0.037	0.253	0.021	0.885	1.037	0.632–1.704
Residence: mixed zones	− 0.267	0.283	0.887	0.346	0.766	0.440–1.334
Residence: rural	0.025	0.185	0.019	0.891	1.026	0.714–1.474
Family relationships						
Family care: no	0.221	0.162	1.852	0.174	1.247	0.907–1.715
Relationships with children: dissatisfied	0.694	0.403	2.960	0.085	2.001	0.908–4.409
Relationships with spouse: dissatisfied	0.648	0.294	4.864	0.027	1.912	1.075–3.401
Relationships with spouse: no spouses	− 0.032	0.323	0.010	0.922	0.969	0.514–1.827
Life satisfaction: dissatisfied	0.576	0.255	5.112	0.024	1.779	1.080–2.931
Health status						
Self-rated health: fair	0.389	0.281	1.915	0.166	1.475	0.850–2.560
Self-rated health: poor	0.449	0.302	2.214	0.137	1.567	0.867–2.833
Health satisfaction: dissatisfied	0.465	0.171	7.388	0.007	1.592	1.138–2.226
Co-morbid conditions: 1	0.147	0.365	0.162	0.688	1.158	0.566–2.368
Co-morbid conditions: 2~	0.436	0.342	1.622	0.203	1.546	0.791–3.024
Heart disease: yes	0.107	0.163	0.427	0.514	1.112	0.808–1.531
Lung disease: yes	0.132	0.203	0.426	0.514	1.141	0.767–1.698
Physical pain: yes	0.331	0.166	3.959	0.047	1.392	1.005–1.928
Work ability: unable to work long hours	0.324	0.182	3.173	0.075	1.382	0.968–1.974
Work ability: unable to work at all	0.134	0.196	0.467	0.494	1.143	0.779–1.679
Lifestyle						
Drinking: yes	0.186	0.174	1.149	0.284	1.205	0.857–1.694
Smoking: yes	0.133	0.184	0.524	0.469	1.143	0.796–1.640
Sleep duration: abnormal	0.443	0.165	7.182	0.007	1.557	1.126–2.152
Stroke treatment: no	-0.179	0.161	1.238	0.266	0.836	0.610–1.146

Further stratified analyses by gender showed that, in males, patients with primary to secondary school education tended to report depressive symptoms compared with those with high school education and above (OR 2.252, 95% CI 1.359–3.730), and patients who were not satisfied with their spouse (OR 2.559, 95% CI 1.116–5.869) as well as health (OR 1.722, 95% CI 1.115–2.661) had a higher likelihood of experiencing depressive symptoms than those who were (Supplementary Table 1). In females, patients aged 60 years or older were less likely to report depressive symptoms than those aged 45–59 years old (OR 0.505, 95% CI 0.285–0.893), and patients without family caregivers (OR 1.720, 95% CI 1.035–2.861) and with abnormal sleep (OR 1.882, 95% CI 1.137–3.116) were more likely to report depressive symptoms (Supplementary Table 2).

## Discussion

Nearly 60% of stroke patients in the study reported experiencing depressive symptoms. In the univariate analysis, significant associations were found between demographic characteristics, family relationships, health status, lifestyle, and depressive symptoms. The results of multivariable analysis further showed that female sex, lower education level, dissatisfaction with the spouse, dissatisfaction with life, dissatisfaction with health, physical pain, and abnormal sleep were significantly associated with depressive symptoms in Chinese stroke patients.

This study showed that the prevalence of depressive symptoms among stroke patients was 57.8%, which was higher than that previously reported in China^24–27^ and other countries^28,29^. In two hospital-based surveys in China^24,25^, the proportions of stroke patients with depression were 41.1% and 31.4%, respectively. This discrepancy is partly due to the use of different diagnostic instruments. In most studies^24,25,27^, depression was clinically diagnosed according to International Classification of Diseases (ICD) codes, whereas our study used the CESD-10, a self-rated scale, to assess depressive symptoms, which might have identified more sub-threshold or mild cases. When different studies used the same diagnostic instrument, the reported prevalence rates were closer. Sit et al. investigated the prevalence of post-stroke depression using the CESD-20 items in Hong Kong, China, and reported a prevalence of 69% and 48% at 48 h and 6 months after stroke, respectively^30^. On another note, ignoring the classification of different stroke types and stroke stages may also result in differences in depression prevalence. However, lower prevalence rates in the United States (23.29%)^28^, Finland, and Sweden (12.8%)^29^ might be due to greater awareness of mental health and more preventive and treatment measures for depression in these countries.

Our analysis showed that female sex was significantly associated with depressive symptoms. This is consistent with previous studies^27,28,31–33^, including two large prospective studies from the United Kingdom and Denmark^32,33^. However, there is still ongoing debate in the literature regarding whether females are more susceptible to post-stroke depression than males. In a systematic analysis by Ryck et al., of 21 studies reviewed, only 7 studies identified female sex as a risk factor for post-stroke depression^34^. According to Wang et al., the association between sex and depression in stroke patients might be influenced by the stage of depression being evaluated. In their study, they observed that female sex was an independent risk factor for post-stroke depression in the acute stroke phase instead of the chronic phase^27^. The results of our univariate and multi-variable analyses showed that stroke patients with lower education levels had a higher probability of having depressive symptoms. Likewise, a cohort study indicated that education level (high school or above) was a protective factor for post-stroke depression at 3 months after stroke event^27^.

Our study revealed that family support and relationships play crucial roles in overcoming depression after a stroke. In the univariate analysis, we found that stroke patients without family caregivers had a higher proportion of being depressed than those with family caregivers. Moreover, we observed that stroke patients who were dissatisfied with their children in the univariate analysis and dissatisfied with their spouse and life in both univariate and multi-variable analyses were more likely to experience depressive symptoms. These findings agree with a population-based study that identified family support as a major protective factor against depression among Chinese elderly^35^. Stroke patients need the support of their family members during the recovery process, especially if they have disabilities and dysfunctions. A well-established family support system can provide life and emotional support to stroke patients and help them deal with psychological stress.

Self-rated health is a subjective measure of an individual’s overall physical, psychological, social, and functional status^36^. In stroke patients, self-rated health can reflect the individual’s adjustment and recovery conditions after a stroke event^37^. Our study reported a significant link between poor self-rated health and depressive symptoms in the univariate analysis. Likewise, Araújo et al. identified emotional functioning as a correlate of an individual’s self-rated health after stroke in the chronic phase^38^. In addition, we also observed a significant association between health dissatisfaction and depressive symptoms in stroke patients. Multi-morbidity is increasingly common and has a bi-directional relationship with depression^39^. Univariate analysis results indicated that depressive symptoms in stroke patients were significantly associated with co-morbid conditions (two or more) and the type of chronic conditions, specifically heart disease and lung disease. Similarly, Jørgensen et al. found that a high level of somatic comorbidity was a significant risk factor for depression in patients with and without stroke. Additionally, their study identified diabetes as an important risk factor for depression^33^. Consistent with previous literature^40^, our study confirmed the association between physical pain and depressive symptoms in Chinese stroke patients. Consistent with previous studies^20^, stroke survivors with inability to work were more likely to be depressed. One possible reason for this is that stroke patients who were unable to work may be more concerned about their economic stability and were prone to depression^41^.

Our univariate and multi-variable analyses revealed a significant association between sleep duration and depressive symptoms. Similar relations between insomnia, sleep disorder, and depression were found in previous studies^24,28^. Unexpectedly, our univariate analysis results showed that stroke patients who were under stroke treatment were more likely to experience depressive symptoms than those who were not, although this association became insignificant in multivariable analysis. This might be because individuals who have more concerns about their disability and functional status are more likely to seek stroke treatment^21^.

The gender-stratified analyses conducted in our study unveiled significant discrepancies in the factors influencing depressive symptoms among males and females. Notably, among males, education level, satisfaction with one's spouse, and satisfaction with one's health status emerged as prominent factors, while among females, age, family caregivers, and sleep patterns appeared to be key contributors. However, it is worth noting that a recent study assessing gender differences in the influence of the five most established risk factors, including functional dependency, a history of pre-stroke depression, social support, stroke severity, and cognitive impairment, found no gender-specific effects^42^. This suggests a wider relevance of these risk factors across genders. Nevertheless, the impact of gender in the context of stroke and depression warrants further investigation and highlights the need for more gender-specific studies in this area.

This study has several implications for health policy and practice. First, our study reported a high prevalence of depressive symptoms (57.8%), suggesting the mental health status of stroke patients is an urgent attention and resolution in China. Second, female and less educated stroke patients were found to be more likely to experience depressive symptoms, highlighting the need for routine screening and assessment of depression among these high-risk groups. Third, our findings underscore the significance of a well-functioning family support system for stroke patients’ mental health and well-being, as demonstrated by the significant correlation between family relationships and depressive symptoms. Fourth, given the correlation between health status and depressive symptoms, our study underscores the importance of pain management and health management in clinical practice in stroke patients. Finally, our study found that lifestyle, particularly sleep duration, was significantly associated with depressive symptoms, suggesting that adjusting sleep habits might improve depression status in stroke patients.

One of the strengths of this study is that it estimated the prevalence and associated factors of depressive symptoms among stroke patients in China using a nationwide dataset. Another strength of this study is that, besides social demographic characteristics and health status, modifiable factors like family relationships and lifestyle were investigated in this study for their potential association with depressive symptoms, which could help in planning interventions. However, our study has several limitations that need to be considered. First, we recognized that our study evaluated depressive symptoms by using CESD-10, rather than diagnosing clinical depression with ICD codes. The use of this method may limit our ability to directly compare our results with previous research that examined diagnosed clinical depression. Second, several prospective studies with large sample sizes showed that stroke severity and pre-stroke depression were strong predictors of depression in stroke patients^32,33^. However, because the assessment of stroke in the CHARLS was mainly based on self-report, and not on detailed diagnostic criteria or specific assessment methods, the CHARLS dataset did not include clinical information about the stroke, such as type (ischemic strokes or hemorrhagic strokes), stage, severity, lesion location, or pre-stroke depression, which were therefore not analyzed. We acknowledge the importance of this issue and stress the need for caution in interpreting our findings given the limitations of the available data. Third, as this study only involved middle-aged and elderly stroke patients in China, the generalizability of our findings may be limited.

Depressive symptoms were highly prevalent in Chinese stroke patients and were associated with various factors related to demographics, family relationships, health status, and lifestyle. Our findings suggest that routine screening and assessment of depression after a stroke is necessary, and that a well-functioning support system, effective health management, and lifestyle modifications may improve depression in stroke patients.

## Methods

### Study design and data source

This is a national cross-sectional survey that focuses on identifying factors associated with depressive symptoms of stroke patients. This study selected samples from the China Health and Retirement Longitudinal Study (CHARLS) 2018 dataset, which includes data from 28 provinces, 150 counties and districts, and 450 urban and rural communities. CHARLS is the first nationally representative health survey of the Chinese elderly population aged 45 years and above, covering information on social, economic, and health status. CHARLS employed a multi-stage stratified Probability Proportional to Size (PPS) method for participant selection. The national baseline survey of CHARLS was conducted in 2011, and follow-up surveys have been conducted every two years since then. As of the fourth wave of data updates in 2018, the number of respondents to CHARLS was 19,816. More details regarding the survey's objectives and methods have been previously published^43^.

In this study, we included a total of 963 patients aged 45 years and older who had self-reported having had a stroke as eligible samples. In addition, it is important to note that our evaluation did not distinguish between ischemic and hemorrhagic strokes due to the reliance on self-report for stroke assessment and the lack of detailed diagnostic criteria in the dataset.

### Depression measurement

Depression was assessed using the 10-item Center for Epidemiological Studies Depression Scale (CESD-10) in CHARLS. The CESD-10 measures the frequency of certain feelings or behaviors in the past week. It consists of 10 questions with four options for each question: rarely or none of the time (< 1 day), some or a little of the time (1–2 days), occasionally or a moderate amount of the time (3–4 days), and most or all of the time (5–7 days). Positive feelings or behaviors are scored as 3, 2, 1, and 0 points respectively, while negative ones are scored as 0, 1, 2, and 3 points. The total score ranges between 0 and 30 points. A higher score indicates more severe depressive symptoms. Following previous studies^44^, we used a cut-off point of 10 points. Respondents with a score of 10 or more were classified as having depressive symptoms and coded as 1, while those with a lower score were considered non-depressed and coded as 0.

### Research variables

Research variables were categorized into four groups: demographics, family relationships, health status, and lifestyle. Demographic variables included age, sex, education level, marital status, and place of residence. Family relationships contained the number of children, family care, relationships with children, relationships with the spouse, and life satisfaction. Health status comprised self-rated health, health satisfaction, chronic comorbidity, physical pain, and work ability. Lifestyle consisted of drinking, smoking, physical activity, social activity, sleep duration (sleep between 5 and 9 hours was considered normal), and stroke treatment. Physical activity was measured primarily based on whether the participant engaged in vigorous, moderate, or mild activities for at least 10 min each week^45,46^. Social activities included interacting with friends, playing board games, helping family, friends, or neighbors who do not live with the participant, going to a club, participating in a community organization, doing voluntary or charity work, caring for sick or disabled adult who does not live with the participant, attending an educational or training course, investing in stocks, using the Internet, and other social activities. Participants who engaged in any of these social activities in the past month were classified into one group and those who did not were classified into another group^47,48^.

### Statistical analysis

We processed the data using SPSS 22.0 (SPSS, Inc., Chicago, USA). Descriptive statistics were conducted to understand the prevalence of depressive symptoms and variable distribution. Then, the Chi-square test was adopted for univariate analysis to explore the associations of depressive symptoms with demographics, family relationships, health status, and lifestyle. Variables with *P*-value < 0.2 in univariate analysis were included in binary logistic regression for multi-variable analysis to further examine these associations. The results of the multi-variable analysis were presented as adjusted ORs and 95% CI. The significant level was set at 5%.

### Ethical aspects

The CHARLS survey was approved by the Biomedical Ethics Review Committee of Peking University, China (approval number: IRB00001052-11015), and informed consent was obtained from all participants before conducting interviews. All research procedures were performed by the Declaration of Helsinki and other relevant guidelines and institutional regulations applied to studies involving human participants.

### Supplementary Information

Supplementary Tables.

## Data Availability

All of the data generated and/or analyzed in this study are available in the [CHARLS] repository [https://charls.charlsdata.com/pages/Data/2018-charls-wave4/zh-cn.html], which is open and free of charge to researchers worldwide. However, to protect the privacy of respondents, users are required to register an account with their real name and institution before accessing the data.
