# Supplementary material for: Analysis of factors associated with depressive symptoms in stroke patients based on a national cross-sectional study
Source: Sci Rep. 2024 Apr 23;14:9268. doi: 10.1038/s41598-024-59837-3 (PMC11035548; doi:10.1038/s41598-024-59837-3)
Supplement: Supplementary file 1 — Supplementary Tables. [file 41598_2024_59837_MOESM1_ESM.docx]

**Supplementary Table 1 Multi-variable analysis of depressive symptoms in male patients**

| **Variable** | **β** | **SE** | **Wald** | ***P-*value** | **OR** | **95% CI** |
| --- | --- | --- | --- | --- | --- | --- |
| Demographics |  |  |  |  |  |  |
| Education: primary to middle school | 0.812 | 0.257 | 9.939 | 0.002 | 2.252 | 1.359 to 3.730 |
| Education: no formal education | 0.620 | 0.391 | 2.510 | 0.113 | 1.859 | 0.863 to 4.002 |
| Relationships with children: dissatisfied | 0.860 | 0.500 | 2.963 | 0.085 | 2.364 | 0.888 to 6.298 |
| Family relationships |  |  |  |  |  |  |
| Relationships with spouse: dissatisfied | 0.940 | 0.424 | 4.920 | 0.027 | 2.559 | 1.116 to 5.869 |
| Relationships with spouse: no spouses | 0.019 | 0.361 | 0.003 | 0.958 | 1.019 | 0.503 to 2.067 |
| Life satisfaction: dissatisfied | 0.618 | 0.353 | 3.059 | 0.080 | 1.855 | 0.928 to 3.706 |
| Health status |  |  |  |  |  |  |
| Self-rated health: fair | 0.240 | 0.355 | 0.458 | 0.498 | 1.272 | 0.634 to 2.549 |
| Self-rated health: poor | 0.528 | 0.380 | 1.933 | 0.164 | 1.696 | 0.805 to 3.573 |
| Health satisfaction: dissatisfied | 0.544 | 0.222 | 6.002 | 0.014 | 1.722 | 1.115 to 2.661 |
| Co-morbid conditions: 1 | 0.128 | 0.454 | 0.080 | 0.778 | 1.137 | 0.467 to 2.768 |
| Co-morbid conditions: 2~ | 0.539 | 0.420 | 1.647 | 0.199 | 1.714 | 0.753 to 3.906 |
| Lung disease: yes | 0.054 | 0.268 | 0.041 | 0.840 | 1.056 | 0.624to 1.785 |
| Physical pain: yes | 0.340 | 0.206 | 2.735 | 0.098 | 1.405 | 0.939to 2.103 |
| Work ability: unable to work long hours | 0.169 | 0.239 | 0.498 | 0.480 | 1.184 | 0.741 to 1.892 |
| Work ability: unable to work at all | -0.111 | 0.253 | 0.194 | 0.660 | 0.895 | 0.545 to 1.469 |
| Lifestyle |  |  |  |  |  |  |
| Sleep duration: abnormal | 0.261 | 0.223 | 1.363 | 0.243 | 1.298 | 0.838 to 2.011 |

The variables age, marital status, place of residence, number of children, family care, hypertension, diabetes mellitus, heart disease, drinking, smoking, physical activity, social activity, and stroke treatment were excluded from the multivariable analysis as their p-values were greater than 0.2 in the univariate analysis.

**Supplementary Table 2 Multi-variable analysis of depressive symptoms in female patients**

| **Variable** | **β** | **SE** | **Wald** | ***P-*value** | **OR** | **95% CI** |
| --- | --- | --- | --- | --- | --- | --- |
| Demographics |  |  |  |  |  |  |
| Age: 60~ | -0.684 | 0.291 | 5.522 | 0.019 | 0.505 | 0.285 to 0.893 |
| Education: primary to middle school | 0.401 | 0.439 | 0.836 | 0.361 | 1.493 | 0.632 to 3.529 |
| Education: no formal education | 0.725 | 0.489 | 2.200 | 0.138 | 2.064 | 0.792 to 5.376 |
| Residence: mixed zones | -0.430 | 0.450 | 0.915 | 0.339 | 0.650 | 0.269 to1.571 |
| Residence: rural | 0.190 | 0.287 | 0.439 | 0.508 | 1.209 | 0.689 to 2.121 |
| Family relationships |  |  |  |  |  |  |
| Number of children: 1-2 | -0.162 | 0.257 | 0.398 | 0.528 | 0.850 | 0.514 to 1.407 |
| Family care: no | 0.543 | 0.260 | 4.371 | 0.037 | 1.720 | 1.035 to 2.861 |
| Relationships with children: dissatisfied | 0.532 | 0.685 | 0.603 | 0.437 | 1.702 | 0.445 to 6.512 |
| Relationships with spouse: dissatisfied | 0.521 | 0.420 | 1.542 | 0.214 | 1.684 | 0.740 to 3.834 |
| Relationships with spouse: no spouses | 0.007 | 0.317 | 0.000 | 0.983 | 1.007 | 0.541 to 1.875 |
| Life satisfaction: dissatisfied | 0.562 | 0.376 | 2.236 | 0.135 | 1.755 | 0.840 to 3.667 |
| Health status |  |  |  |  |  |  |
| Self-rated health: fair | 0.753 | 0.476 | 2.501 | 0.114 | 2.123 | 0.835 to 5.400 |
| Self-rated health: poor | 0.420 | 0.512 | 0.673 | 0.412 | 1.522 | 0.558 to 4.150 |
| Health satisfaction: dissatisfied | 0.341 | 0.277 | 1.521 | 0.217 | 1.407 | 0.818 to 2.420 |
| Co-morbid conditions: 1 | -0.028 | 0.656 | 0.002 | 0.966 | 0.973 | 0.269 to 3.521 |
| Co-morbid conditions: 2~ | -0.049 | 0.607 | 0.006 | 0.936 | 0.952 | 0.290 to 3.130 |
| Heart disease: yes | 0.384 | 0.248 | 2.396 | 0.122 | 1.468 | 0.903 to 2.386 |
| Lung disease: yes | 0.273 | 0.321 | 0.721 | 0.396 | 1.313 | 0.700 to 2.464 |
| Physical pain: yes | 0.424 | 0.297 | 2.045 | 0.153 | 1.528 | 0.854 to 2.734 |
| Work ability: unable to work long hours | 0.500 | 0.282 | 3.142 | 0.076 | 1.649 | 0.949 to 2.866 |
| Work ability: unable to work at all | 0.564 | 0.316 | 3.185 | 0.074 | 1.758 | 0.946 to 3.266 |
| Lifestyle |  |  |  |  |  |  |
| Sleep duration: abnormal | 0.632 | 0.257 | 6.042 | 0.014 | 1.882 | 1.137 to 3.116 |
| Stroke treatment: no | -0.287 | 0.250 | 1.312 | 0.252 | 0.751 | 0.462 to 1.226 |

The variables marital status, hypertension, diabetes mellitus, drinking, smoking, physical activity and social activity were excluded from the multivariable analysis as their p-values were greater than 0.2 in the univariate analysis.
